# Supplementary material for: A Decision Aid for Postpartum Adolescent Family Planning: A Quasi-Experimental Study in Tanzania
Source: Int J Environ Res Public Health. 2023 Mar 10;20(6):4904. doi: 10.3390/ijerph20064904 (PMC10049540; doi:10.3390/ijerph20064904)
Supplement: Supplementary file 1 [file ijerph-20-04904-s001.zip › File S2 Informed Assent Form for Pregnant Adolescents between 10-14 years.pdf]

S2: Informed Assent Form for Pregnant Adolescents between 10-14 years

ID – NO			

Title of the Study: Effect of the Family Planning "Green Star" Decision Aid in the Choice for Postpartum Family Planning Among Adolescent Mothers in Tanzania: Facility Based Quasi-Experimental Design

Investigator Name: Stella E. Mushy; Mobile number: +255 715 796 077

Institution Name: St. Luke's International University, Tokyo, Japan

Supervisor's Name: Shigeko Horiuchi, Department of Midwifery

Note: This study was granted permission to be carried out from the head of the research institution of St. Luke's International University, Tokyo, Japan; Muhimbili Institutional Review Board of University of Health and Allied Sciences (MUHAS), Dar es Salaam, Tanzania; and the National Institute of Medical Research, Dar es Salaam, Tanzania.

Background and Rationale of the Study: Adolescent pregnancy remains a significant national problem and is a critical health and social priority in Tanzania. Recent data indicate huge unmet needs for postpartum family planning methods among adolescent mothers, thus they experience a higher risk of subsequent unplanned and unwanted pregnancies than older women. The majority of research and interventions in adolescent sexual and reproductive health globally focused on prevention or delay of first pregnancy amongst adolescents. The present study is exceptional in that it addresses an event further overlooked in a subgroup of adolescent mothers and looks to improve their education and socio-economic achievement by helping them utilize the available long-acting reversible family planning methods immediately after birth. To our knowledge, this is the first study

targeting adolescent mothers and aiming to help them prevent the occurrence of subsequent pregnancies (with the inherent risks and long-term challenges) by this subgroup.

Purpose of the Study

The purpose of this study is to evaluate the effect of the family planning "Green Star" decision aid on reducing decision making conflict, improving satisfaction rate and utilizing long-acting reversible family planning methods among pregnant adolescents. The name green star stands for Nyota ya Kijani meaning artificial family planning methods that any woman of a reproductive age can use to space or limit the number of children one should have. The tool has evidence-based information on the benefits, side effects, complications, fertility return, and satisfaction and continuation rates of long-acting reversible family planning methods to help women understand each method correctly so that they can clearly decide on the option to use a particular method after birth.

Study procedure

On agreeing to participate in the study, participants from Kisarawe Hospital will be assigned to the control group while participants from Mkuranga Hospital will be assigned to the intervention group. Details of the services the participant will receive in each group are stated below.

Intervention group

Each participants receive a minimum of three visits at 28, 32, 36/38 weeks before giving birth. The first education session will be the routine FP education being offered in every visit. This will then be followed up by an individual face to face FP counselling education that will take 40 minutes to one hour for her to clearly understand the information

presented in the tool. The tool will be offered by a trained healthcare provider to her at each visit as per the contents available in the "Green Star DA" education material. She will receive the "Green Star" DA and they will be allowed to carry it home for further reading. In case of any question (s), a note is written down at the end page for clarification in the next visit. The decision aid has 10 pages.

Control group

You child will only receive a routine/usual family planning counseling offered at each ANC visit. Expect her to receive a minimum of three education sessions at 28, 32, and 36/38 weeks before she gives birth.

Study participants

The intended study participants are pregnant adolescents who; i) are aged between 10-19 years, ii) have 28 – 34 gestation weeks, iii) are planning to deliver and attend postnatal services at the hospital they are attending ANC services, iv) are willing and consented to participate in a study, v) are mentally and physically well/disabled, and vi) are able to communicate in Kiswahili language.

Risks/Discomfort

There is no anticipated risk by involving in the study. However, you may experience some concern or worries while responding to questions. It will take 20-30 minutes of her time to answer all the questions.

Benefits

There is no direct benefit in participating in this study. However, the information that is obtained will help to improve the Green Star decision making that will be used to educate adolescents on the importance of using long-acting reversible family planning methods

so as to prevent the occurrence of unplanned and unwanted subsequent pregnancies during teen ages.

Compensation for participation in the study

You will not be paid in any way for participating in this study but you will be given a set of delivery kit as an appreciation for your participation as well as ensuring clean and safe delivery. The study is funded by the Japan Society for the Promotion of Science Core-to-core program (2018-2021).

Voluntary

Taking part in this research is completely voluntary or your choice. You are allowed to refuse participating or you can withdraw in this study at any time, even if you have already given consent. Refusal to participate or withdraw from the study will not involve any penalty. However, the information you provided to us will be retained and analyzed when we find the information falls within the scope of the analysis we are looking at.

Confidentiality

1) The results you give will be kept strictly confidential, and used only for research purposes such as presentations in conferences and publications.

2) Your identity will be hidden in as far as the law allows. The research protocol and other materials can be accessed at the request of the research subject to avoid disclosing information of other participants.

3) Your information will be stored in a locker with a key at St Luke's International University, second building, 7th floor.

4) Your information will be kept for five years after the end of study then we will destroy them with a shredder

5) Researchers and funders of this study do not have any conflict of interest

In case of any question, where you need further clarification you can contact Stella E. Mushy Tel: +255 715 796 077. For issues regarding ethics of this study please contact the Director of Research and Publications Committee at MUHAS, Mr. Bruno F. Sunguya, Tel: +255 685 217 272, P.O. Box 9653, Dar es Salaam.

STATEMENT OF CONSENT

Has described to me what is going to be done, the risks, the benefits involved and my rights regarding this study. I understand that my decision to participate in this study will not alter my usual medical care. In the use of this information, my identity will be concealed. I am aware that I may withdraw at any time. I understand that by signing this form, I do not waive any of my legal rights but merely indicate that I have been informed about the research study in which I am voluntarily agreeing to participate. A copy of this form will be provided to me.

Signature of the participant …………………… Age ………… Date ………………

Signature of the parent/guardian……………….. Date………………….

Signature of Researcher …………………… Date ………………………..
