# Supplementary material for: A Decision Aid for Postpartum Adolescent Family Planning: A Quasi-Experimental Study in Tanzania
Source: Int J Environ Res Public Health. 2023 Mar 10;20(6):4904. doi: 10.3390/ijerph20064904 (PMC10049540; doi:10.3390/ijerph20064904)
Supplement: Supplementary file 1 [file ijerph-20-04904-s001.zip › File S3 Participants Withdrawal Form (1).pdf]

S3: Participants Withdrawal Form

ID – NO				

Tittle: Effect of the Family Planning "Green Star" Decision Aid in the Choice for Postpartum Family Planning Among Pregnant Adolescents in Tanzania: Facility Based Quasi-Experimental Design

(Only to be completed by study staff in case of withdrawal)

Please note the participant's agreement to the following options if the participant has withdrawn from the study activities

Options to review with the participants at the time of withdraw

Check ☒ all that are applicable

☐ I will continue to return for follow-up study visits as planned and will take part in study activities (as described in the main consent) until the study is closed; however, I do not allow my information to be used anywhere.

☐ I do not agree to continue with the study, thereby I withdraw my consent for all further study-related activities. Whatever the information you received from me can be further used if needed.

☐ I do not agree to continue with the study, thereby I withdraw my consent from the intervention and I do not allow my information to be used anywhere.

________________________ ____________________ _______________

Signature of Study Staff | Printed Name | Date of Signature

____

Study participant ID Number
