# Supplementary material for: A Decision Aid for Postpartum Adolescent Family Planning: A Quasi-Experimental Study in Tanzania
Source: Int J Environ Res Public Health. 2023 Mar 10;20(6):4904. doi: 10.3390/ijerph20064904 (PMC10049540; doi:10.3390/ijerph20064904)
Supplement: Supplementary file 1 [file ijerph-20-04904-s001.zip › File S4 Ethical Approval Document from St. Lukeüfs.pdf]

St. Luke's International University

This hospital is a living organism designed to demonstrate in convincing terms the transmuting power of Christian love when applied in relief of human suffering.

19- Mar -2021

This is to certify that the research titled "Effect of the family Planning "Green Star" Decision Aid in the Choice for Postpartum Family Planning Among Adolescent Mothers in Tanzania:Facility Based Quasi-Experimetal Design" (Approval number 20-A091) has been approved by St.Luke's International University Research Ethics Committee.

The original document of approval written in Japanese is attached for your reference.

Sincerely,

Yaeko Kataoka

YAEKO KATAOKA RN.PHN.NM.PhD.

chairperson of the committee

St. Luke's International University
9-1 Akashi-cho, Chuo-ku Tokyo
104-8560 Japan
telephone 03-3541-5151
facsimile 03-3544-0649
