# Supplementary material for: A Decision Aid for Postpartum Adolescent Family Planning: A Quasi-Experimental Study in Tanzania
Source: Int J Environ Res Public Health. 2023 Mar 10;20(6):4904. doi: 10.3390/ijerph20064904 (PMC10049540; doi:10.3390/ijerph20064904)
Supplement: Supplementary file 1 [file ijerph-20-04904-s001.zip › File S5 Ethical Approval Document from Muhimbili.pdf]

MUHIMBILI UNIVERSITY OF HEALTH AND ALLIED SCIENCES

OFFICE OF THE DIRECTOR OF RESEARCH AND PUBLICATIONS

P.O. Box 65001
DAR ES SALAAM
TANZANIA
Web: www.muhas.ac.tz

Tel G/Line: +255-22-2150302/6 Ext: 1016
Direct Line: +255-22-2152489
Telefax: +255-22-2152489
E-mail: drp@muhas.ac.tz

Ref. No.DA.282/298/01.C/ 04th March, 2020

MUHAS-REC-1-2020-076

Stella Emmanuel Mushy,
Department of Community Health Nursing,
School of Nursing,
MUHAS

RE: APPROVAL FOR ETHICAL CLEARANCE FOR A STUDY TITLED "THE GREEN STAR, DECISION AID ON A LONG ACTING REVERSIBLE FAMILY PLANNING METHOD UPTAKE BY ADOLESCENT MOTHERS IN TANZANIA"

Reference is made to the above heading.

I am pleased to inform you that the Chairman has on behalf of the University Senate, approved ethical clearance of the above mentioned study, on recommendations of the Senate Research and Publications Committee Meeting.

The validity of this ethical clearance is one year effective from to **27th February, 2020 26th February, 2021.** You will therefore be required to apply for renewal of ethical clearance on a yearly basis if the study is not completed at the end of this clearance.

You will be expected to provide adverse events report where applicable, six monthly progress reports and a final project report upon completion of your study.

DIRECTOR
Research &
Publications
Box 65001

Dr. Bruno Sunguya
Ag. Chairperson, Senate Research and Publications Committee

Scanned by CamScanner
