# Supplementary material for: A Decision Aid for Postpartum Adolescent Family Planning: A Quasi-Experimental Study in Tanzania
Source: Int J Environ Res Public Health. 2023 Mar 10;20(6):4904. doi: 10.3390/ijerph20064904 (PMC10049540; doi:10.3390/ijerph20064904)
Supplement: Supplementary file 1 [file ijerph-20-04904-s001.zip › File S6 Age of Consent Legal Review.pdf]

AGE OF CONSENT: LEGAL REVIEW
TANZANIA COUNTRY REPORT

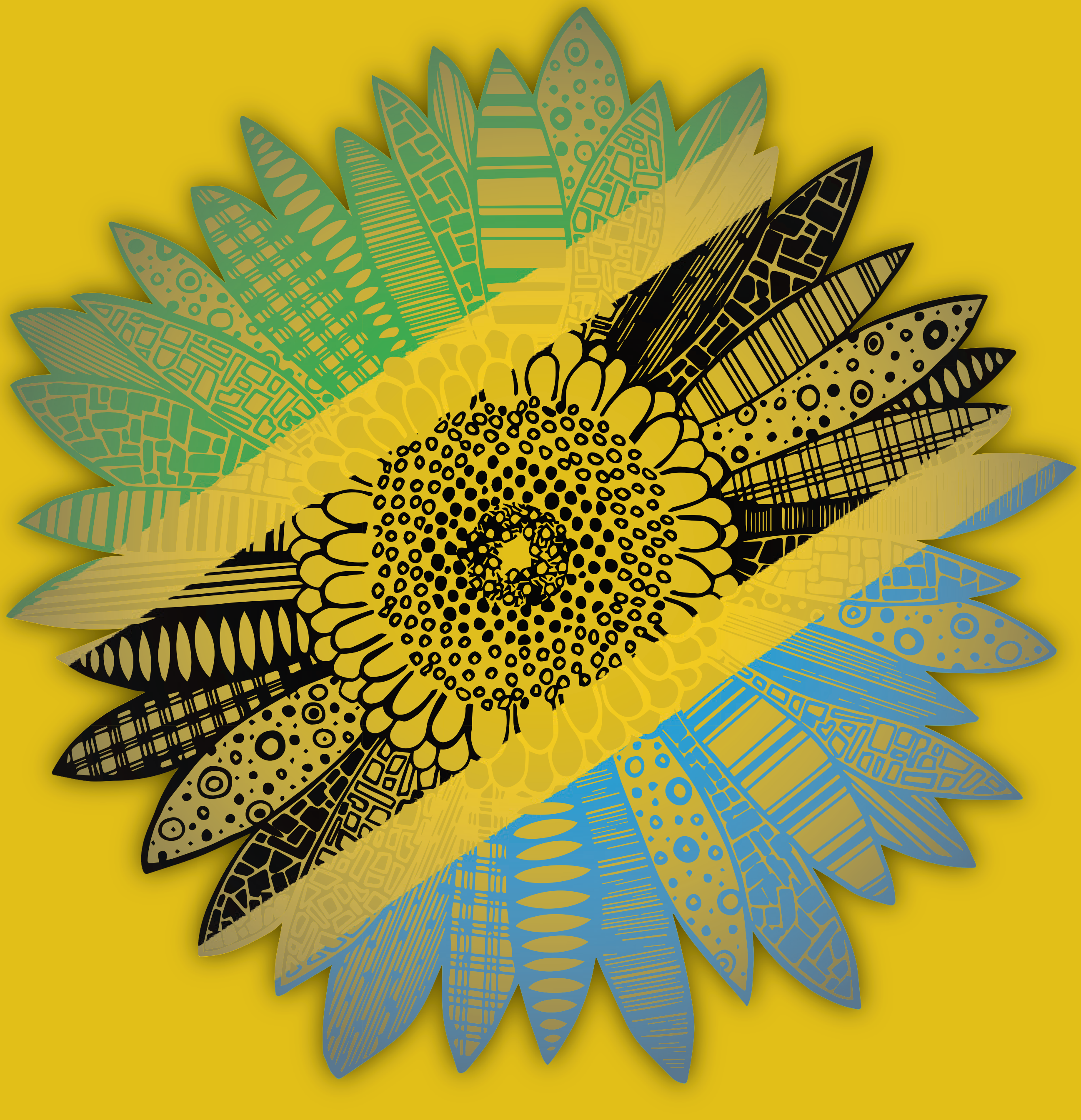

FOREWORD

There is one barrier to health access and a major impediment to national and regional development across the globe that could be removed within a few days if only the policy decisions would be made. The removal of this barrier would promote gender equality, help end child marriage, reduce teenage pregnancy and girl dropout rates from secondary school, lower HIV incidence rates, and achieve the prerequisite for the demographic dividend. Let me explain.

As the Sustainable Development Goals (SDGs) become the world's compass towards development and a better life for all people on the planet, sub-Saharan Africa faces a potential demographic disaster that could undermine many of the SDGs.

Many sub-Saharan countries have some of the highest fertility rates in the world which, if unaltered, will see those countries' population, rising from 17 million to 43 million by 2050. Another country's population that is currently at 27 million will more than double to 65 million by 2050, with yet another going from 53 million to 137 million. Not one of these countries will have reached the population turnaround required as an inescapable prerequisite for achieving a demographic dividend by 2050.

With increases like these on the same available land area, with the same or less access to drinking water, potentially less arable land available due to global warming, and services and GDP increasing at a far slower rate than population, it will be impossible to achieve the SDGs and improbable to even maintain the human development index achieved thus far by 2017. These are not scenarios or 'maybes'. This is the inevitable future of the region unless something significant, such as the fertility rates, changes. Slowing and reversing population acceleration is essential to development in sub-Saharan Africa.

And yet, the world has a dark and shameful history of 'population control', legislating women's rights to decide on having children away and forcing and coercing sterilisation. These restrictions on agency and autonomy have no place in a democratic, sustainable development world in the 21st century and the vast majority of nations achieving the demographic dividend have not resorted to such dark methods.

Increasing gender equality, keeping girls in safe schools for longer, reducing sexual and gender based violence, and increasing the sexual and reproductive health knowledge of young people are also factors. And of course, it is the increased access to modern contraceptives and women making choices about their own reproductive health that have turned the tide.

So this research report turns its attention to this access to modern contraceptive methods, as well as all other sexual and reproductive health (SRH) services. There are many barriers for adolescents around the globe - the majority of citizens in many countries – to access SRH services. Distance from services, lack of transport, lack of available commodities, poverty, inherited beliefs, harmful practices, and hostile staff attitudes can all be barriers to access. But only one barrier is self-inflicted by countries. And this barrier could disappear tomorrow, creating massive access for adolescents. It is the barrier of legal restrictions on adolescents' access to SRH. The majority of countries in the region have placed arbitrary legal barriers to adolescent access by creating policies or laws with a number of parental consent and other restrictions, preventing adolescents from initiating their own healthcare.

In some cases, these have been gradually added to laws or policies with well-intentioned reasons to protect adolescents. In many cases, however, they reflect an idealised view of what older people think younger people should or should not be doing. Primarily, these idealised views revolve around sex. Older people, sometimes forgetting their own youth, think that the best protection for younger people is to abstain from sex until they are, perhaps 16, or even 18. Reality is, in many cases different, and by ignoring reality and locking adolescents out of the health system when it comes to sexual and reproductive health and rights (SRHR), adults are doing irreparable damage to their children and other children, as well as directly contributing to a looming demographic disaster for their nation.

Because this seems so obvious, recent global guidelines have called on countries to examine their current policies and consider revising them to reduce age-related barriers to access and uptake of all SRH services. To assist in this process, the SRHR Africa Trust (SAT) in collaboration with the Thomson Reuters Foundation, 20 global law firms, and UNICEF, has reviewed Age of Consent laws in 22 countries: Botswana, Kenya, Ivory Coast, Malawi, Morocco, Nigeria, South Africa, Swaziland, Tanzania, Zambia, Zimbabwe, Brazil, Jamaica, India, Indonesia, Thailand, Vietnam, Canada, United Kingdom, France, Sweden, and Ukraine. The range of countries was chosen to highlight a spread of geography, cultures, development status, nonachievement, in-transition and achievement of the demographic dividend, and adolescent health outcomes. Inaddition, SAT and UNICEF also undertook an Ethical, Cultural and Social review in nine countries (seven being from the 22 countries above, plus two others - Uganda and Philippines) to look at ethical cultural and social barriers impacting adolescent access to health and services including SRH services.

The data revealed some game-changing recommendations apparent to SAT:

1. Desexualise SRHR services and treat them as you would any other health system access;
2. Base both access and restriction decisions on child and adolescent development, competence and maturity to make decisions about their own health;
3. Do not make the age of sexual consent older than 16 years, and do not distinguish between young men and young women;
4. Have statutory rape provisions in place to protect adolescents from predatory adults;
5. Put ‘close in age’ exceptions to the age of sexual consent in place so that young adolescents having consensual sex with one another do not have to go to jail – a travesty affecting young men aged 13-16 years.

We offer this report and its insights to policymakers, legislators, development agencies, international cooperating partners, and activists wherever they may be. A thinking and action tool is currently being designed to accompany this report. If you wish to receive it or an electronic copy of this report, please send an e-mail to ageofconsent@satregional.org. Please also feel free to share ways in which you may have used this report or the action tool to create positive change with us and with one another.

Our hope is that these resources will create dialogue and action that will remove all legal barriers to adolescent access to SRH services and allow attention and resources to focus on the remaining and more intractable barriers.

Jonathan Gunthorp

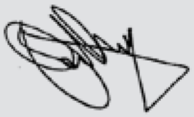

Executive Director - SAT

ACKNOWLEDGEMENT

The SRHR Africa Trust (SAT) wishes to acknowledge, the individuals, organisations and law firms that contributed to this report through their, expertise, co-operation, and hard work.

Special thanks go to Asyla Attorneys Law Firm in Tanzania that provided pro bono legal services to assist SAT with the underlying research for the review on the Age of Consent Legal Review in Tanzania, working with Arnold & Porter Kaye Scholer LLP, and in particular to Catherine Young for coordinating the legal review in all the participating countries.

SAT also wishes to thank civil society organisations and partners who attended the Age of Consent Validation Meeting that met to discuss and validate the draft Advocacy Toolkit. The meeting critical reviewed the draft Advocacy Toolkit, analysing the data collected for its accuracy and merits.

SAT is grateful to UNICEF for co-funding the review of the Age of Consent laws, policy frameworks, Ethical, Social, and Cultural (ESC) impacts on sexual reproductive health and rights (SRHR) and HIV.

Last but not least, SAT thanks TrustLaw at the Thomson Reuters Foundation for their continued collaboration with SAT on health and rights and for brokering the partnerships between SAT and the law firms. TrustLaw is the Thomson Reuters Foundation’s global pro bono legal programme, connecting law firms and corporate legal teams around the world with high impact NGOs and social enterprises working to create social and environmental change.

ASYLA
ATTORNEYS

DISCLAIMER

This legal review report and the information it contains is provided for general informational purposes only.

It has been prepared as a work of comparative legal review only and does not represent legal advice in respect of the laws of Tanzania. It does not purport to be complete or apply to any particular factual or legal circumstances. It does not constitute, and must not be relied or acted upon as legal advice or create an attorney-client relationship with any person or entity.

Neither Asyla Attorneys, the SRHR Africa Trust, nor the Thomson Reuters Foundation accept responsibility for losses that may arise from reliance upon the information contained in this review note or any inaccuracies therein, including changes in the law since the review commenced in February 2016. Legal advice should be obtained from legal counsel qualified in the relevant jurisdiction(s) when dealing with specific circumstances.

Neither Asyla Attorneys, nor any of the lawyers at Asyla Attorneys, the SRHR Africa Trust, nor the Thomson Reuters Foundation is holding itself, himself or herself out as being qualified to provide legal advice in respect of any jurisdiction as a result of his or her participation in or contributions to this legal review report.

TANZANIA

AGE OF CONSENT LEGAL REVIEW

SRHR Africa Trust
GENDER EQUAL YOUTH HEALTH

ACRONYMS

AIDS	Acquired Immune Deficiency Syndrome
ANC	Antenatal Care
ART	Antiretroviral Therapy
EHP	Essential Health Package
GDP	Gross Domestic Product
HCT	HIV Counselling and Testing
HIV	Human Immunodeficiency Virus
HPV	Human Papillomavirus
MMC	Medical Male Circumcision
MPR	Multiple-perpetrator Rape
MSP	Multiple Sexual Partners
PLWHA	People Living with HIV/AIDS
PLWHIV	People Living with HIV
PEP	Post-exposure Prophylaxis
PrEP	Pre-exposure Prophylaxis
SRHS	Sexual and Reproductive Health Services
UNAIDS	Joint United Nations Programme on HIV/AIDS
UNICEF	United Nations International Children’s Fund
UNFPA	United Nations Population Fund
WHO	World Health Organization
YFS	Youth-friendly Services

CONTENT PAGE

FOREWORD		i
ACKNOWLEDGEMENT		iii
DISCLAIMER		iii
ACRONYMS		vi
EXECUTIVE SUMMARY		viii
Chapter One:	Introduction	1
Chapter Two:	Age of Consent to sexual intercourse	3
Chapter Three:	Age of Consent and access to contraception services and commodities	5
Chapter Four:	Age of Consent and HIV testing	6
Chapter Five:	Age of Consent on Antiretroviral Therapy	7
Chapter Six:	Age of Consent and access to Pre-exposure Prophylaxis (PrEP) and Post-exposure Prophylaxis (PEP)	8
Chapter Seven:	Age of Consent and access to safe abortions and/or postabortion care	10
Chapter Eight:	Age of Consent on access to Antenatal Care (ANC)	11
Chapter Nine:	Access to HPV vaccines and cervical cancer screening and treatment	12
Chapter Ten:	Recommended intervention on legal and policy framework	13
Annex		15

EXECUTIVE SUMMARY

The Age of Consent to sexual intercourse in Tanzania is 18 years for females and there is not stipulated limit for males. Sexual intercourse with a woman under the age of 18 years is illegal. However, a man may have sexual intercourse with a female aged 15-17 years if she is his wife. Male gay sex is criminalised.

The law is silent on access to contraception services, commodities and emergency contraception, therefore, there are no age restrictions so parental consent is not a legal requirement. In the absence of any legislation, reference is made to Common Law (as provided in Section 2 of the Judicature and Applications of Laws Act). However research shows that the youngest age of females in the United Republic of Tanzania who use contraceptives is 15 years of age, although 15 was the youngest age that was surveyed, so there may be women younger than 15 using contraceptives. Due to the absence of any explicit rules, there is no law that prevents the usage of contraception at any age and this is, therefore, the default legal position of this matter and parental consent is not required.

The Age of Consent for HIV testing in Tanzania is 16 years and any persons under 16 years requires parental consent to access HIV testing. The law does not stipulate the age at which adolescents' HIV status can be reported directly to them. The results are confidential and results are communicated to the parent or guardian for a person under 18 years of age. The National AIDS Policy of 2001 Tanzania provides the legal and policy framework for the provision of ART. There are no age restrictions, so parental consent is not a legal requirement to access ART.

The country does not have any age restrictions, so parental consent is not a legal requirement.

There is an existing legislation which enables access to HIV Post-exposure Prophhylaxis (PEP) by healthcare practitioners. The law is silent on Pre-exposure Prophylaxis (PrEP), however, all HIV/AIDS infected young people should be treated as per the rights and obligations that they are entitled to which also includes means to access education, basic healthcare and livelihood services among others.

Abortion is illegal in Tanzania, only abortions that are performed to preserve the mother's life are legal. Antenatal Care (ANC) is accounted for in the policy to reduce maternal deaths. It does not deal with the Age of Consent.

Chapter One: Introduction

The world's attention has moved beyond HIV and AIDS, yet it remains the leading killer of adolescents in Southern Africa, presenting a major impediment to healthy communities and a significant factor in the health and status of women and girls in the region.

According to a report by the Joint United Nations Programme on HIV/AIDS (UNAIDS) on prevention of HIV (2013), Eastern and Southern Africa is home to half the world's population living with HIV, even if the region is only 5% of the world's population. Today the region continues to be the most infected and affected by the HIV/AIDS epidemic, with 48% of the world's new HIV infections among adults, 55% among children, and 48% of AIDS-related deaths.

Research conducted in many parts of the world shows that several legal and human rights barriers hinder access to and uptake of HIV services for adolescents. Key among these barriers are Age of Consent legislation and policies. Recent global guidelines have, therefore, explicitly called on countries to examine their current policies and consider revising them to reduce age-related barriers to access and uptake of the overall SRH services, HIV Counselling and Testing (HCT) and linkages to prevention, treatment, and care.

HIV is the most severe in the Southern Africa sub-region which includes Botswana, Lesotho, Malawi, Mozambique, Namibia, South Africa, Swaziland, Zambia, and Zimbabwe, with each having an adult HIV prevalence of over 10%. UNAIDS reports that Swaziland has the highest HIV prevalence rate in the world (26%), followed by Botswana (23.4%).

In 2011, there was an estimated 1.2 million adolescents 10-19 years old living with HIV, more than half of all HIV positive adolescents globally Eastern and Southern Africa now have 10.5 million children who have lost one or both parents to AIDS.

The risk of becoming infected with HIV is higher for girls and young women and currently, HIV prevalence among young women aged 15-24 years stands at 4.8%, which is two and a half times higher than among men of the same age. In Swaziland, 15.6% of young women are HIV positive, compared to 6.5% of young men.

All in To #EndAdolescentAIDS was launched as a global platform for action and collaboration for social change in February 2015 by global leaders and civil society partners. This global agenda provides an opportunity for ensuring that existing and new strategies and programmes on HIV prevention, treatment and care are effectively implemented to benefit young people. These include the 2012 guidance on Pre-exposure Oral Prophylaxis (PrEP) for serodiscordant couples, men, and transgender women who have sex with men at high risk of HIV as well as the 2013 guidance for HIV HCT and care for adolescents living with HIV and young key population policy briefs.

All in To #EndAdolescentAIDS works through four workstreams. As part of workstream 1, global partners have prioritised joint action to take stock of the current situation in countries with respect to Age of Consent for services including HIV prevention by carrying out a systematic desk review of Age of Consent laws and a mapping of the processes led and outcome of reforms undertaken by countries to address this barrier. Through the All In To #EndAdolescentAIDS, many agencies, and global partners including the United Nations Development Programme (UNDP), the Joint United Nations Programme on HIV/AIDS (UNAIDS), the PACT and AIDS Alliance have already initiated actions on data collection to inform their policy advocacy efforts globally and specifically within the 25 focus countries. This review will inform the development of a source or toolkit for learning on this theme and capacity building of experts and policymakers worldwide.

Why Age of Consent review?

Several global bodies, including UNICEF and WHO, are currently undertaking a number of initiatives to change the global guidelines with regard to adolescent access to a range of HIV prevention services, including Age of Consent and informed consent, confidentiality, availability, accessibility, and acceptability. Adherence and quality must inform these services.

The SRHR Africa Trust (SAT) in collaboration with UNICEF worked with participating local law firms, and a consultant to conduct the Age of Consent research. The three areas of the research included the following:

1. Age of Consent legal review
2. Ethical, Social, Cultural (ESC) desktop review
3. Youth attitudes survey

Therefore, the main goal of the project is to conduct research on "Age of Consent" laws, regulations, and policies, as well as ESC factors, in relation to adolescents. This entailed collecting country experiences to address Age of Consent laws / polices and ESC factors that serve as potential barriers to adolescents accessing SRH services, including HIV prevention, care, and treatment. The research explored implications for expanding SRHR services and HCT in adolescents aged 10–19 years.

Methodology

The Tanzania legal review was prepared by SAT and is based on research conducted by the Asyla Attorneys Law Firm in Tanzania. The legal review focuses on the laws and policy support around the Age of Consent in relation to the various aspects relating to SRHR. The review looks at all the relevant national laws (including nationally recognised customary or religious laws), regulations, and policies exploring the ages for girls and boys separately, where relevant, including where contradictions in laws, policies, and regulations on these issues exist.

The review specifically looked at the following areas:

1. Age of Consent to sexual intercourse including the age for statutory rape
2. Age of Consent to access modern contraceptives, with and without parental consent
3. Age of access to emergency contraceptives with and without parental consent
4. Policy framework and legislation enabling or disenabling access to Pre-exposure Prophylaxis (PrEP), including Age of Consent, with and without parental consent
5. Policy framework and legislation enabling or disenabling access to Post-exposure Prophylaxis (PEP); including Age of Consent, with and without parental consent
6. Policy framework and legislation enabling or disenabling access to safe abortions and postabortion care, including Age of Consent, with and without parental consent
7. Policy framework and legislation enabling or disenabling access to Antenatal Care (ANC), including Age of Consent, with and without parental consent
8. Policy framework and legislation enabling or disenabling access to HPV vaccines and cervical cancer screening and treatment, including Age of Consent, with and without parental consent
9. Age of Consent to access HIV testing without parental consent.

Chapter Two: Age of Consent to sexual intercourse

The Age of Consent to sexual intercourse is 18 years for women, except that it is lawful for a man to have sex with his wife who may be younger than 18 years, with or without her consent, provided she is at least 15 years old. For a wife over the age of 15 years, it is immaterial to seek consent. However, if they are separated, then consent must be obtained.

Section 13 of the Law of Marriage Act contains the words 'apparent age', this is simply the age that is recognised by the law (15 years). This age was chosen because a majority of females in the United Republic of Tanzania, have reached puberty at the age of 15 years.

The law is not specific on the Age of Consent for men. The following Act may elaborate on this the position:

Legislation on Age of Consent to sexual intercourse

Sexual offence special provisions Act of 1998:

Section 5 Rape (Section 5 replaces Section 130 of the Penal Code Cap 16)

2. *A male person commits the offence of rape if he has sexual intercourse with a girl or woman under circumstances falling under any of the following descriptions:*
   e. *with or without her consent when she is under eighteen years of age, unless the woman is his wife who is fifteen or more years of age and is not separated from the man.*

Law of Marriage Act Cap 29 RE: 2002:

Section 13 Minimum Age

1. *No person shall marry who, being male, has not attained the apparent age of eighteen years or, being female, has not attained the apparent age, which means exactly the age of fifteen years.*
2. *Notwithstanding the provisions of subsection (1), the court shall, in its discretion, have the power, on application, to give permission for a marriage where the parties are, or either of them is, below the ages prescribed in subsection (1) if– (a) each party has attained the age of fourteen years; and (b) the court is satisfied that there are special circumstances which make the proposed marriage desirable.*
3. *A person who has not attained the apparent age of eighteen years or fifteen years, as the case may be, and in respect of whom the leave of the court has not been obtained under subsection (2), shall be said to be below the minimum age for marriage.*

Definition of statutory rape

The offence of rape is defined by the absence of consent. Statutory rape refers to sexual relations involving someone below the 'Age of Consent' (i.e., someone who cannot consent). People below the Age of Consent cannot legally consent to having sex. This means that by having sex with them, by definition, violates the law.

Legislation and policy framework on statutory rape

Category of regulation is legislation. The relevant section is quoted as follows:

Penal Code Cap 16 RE: 2002

Section 130.-(l) It is an offence for a male person to rape a girl or a woman.

2. *A male person commits the offence of rape if he has sexual intercourse with a girl or woman under circumstances falling under any of the following descriptions:*
   a. *not being his wife, or being his wife who is separated from him, without her consenting to it at the time of the sexual intercourse;*
   b. *with her consent where, the consent ... has been obtained by the use of force threats or intimidation or by putting her in fear of death or of hurt while she is in unlawful detention.*

Exceptions on Age of Consent - For example 'gay sex'

In Tanzania, there is no explicit exception on gay sex. Any male person found to be involved in gay sex is liable for imprisonment for five years. The legislature states as follows:

Penal Code Cap 16 RE: 2002
Section 157:

Any male person who, whether in public or private, commits any act of gross indecency with another male person to commit any act of gross indecency with him, or attempts to procure the commission of any such act by any male person, with himself or with another male person, whether in public or private, commits an offence and liable to imprisonment for five years.

Chapter Three: Age of Consent and access to contraception services and commodities

The law is silent on the age at which a young person may access contraceptive services. In the absence of any legislation, reference is made to Common Law (as provided in Section 2 of the and Applications of Laws Act). However, research shows that the youngest age of females in the United Republic of Tanzania who use contraceptives is 15 years of age, although 15 years was the youngest age that was surveyed, so there may be women younger than 15 years using contraceptives. Due to the absence of any explicit rules, there is no law that prevents the usage of contraception at any age and this is, therefore, the default legal position of this matter and parental consent is not required.

This is inconsistent with the Age of Consent to sexual intercourse as described in Chapter 1. There is no general Age of Consent to medical treatment.

Legislation and policy framework

The Tanzania Demographic Health Survey of 2010 shows that 29% of all women, 34% of currently married women, and 51% of sexually active unmarried women aged 15-49 years are using a contraceptive method. The 2008 HIV & AIDS (prevention and control act) (HIV & AIDS ACT.)

Access to emergency contraceptives

There are no laws or policies regulating access to emergency contraceptives. Therefore, due to the absence of any explicit rules relating to emergency contraceptive, the research can safely conclude that a person may access emergency contraceptives at any age and without parental consent.

Chapter Four: Age of Consent and HIV testing

The Age of Consent for HIV testing is 16 years in Tanzania. Testing of persons under the age of 16 must be carried out with the consent of their parents or legal guardians. The age of majority in the United Republic of Tanzania is 18 years. Adolescents aged 16-17 years are categorised as 'mature minors' and, therefore, can give consent themselves. This Age of Consent only extends to HIV testing.

Legislation and policy framework on HIV testing

HIV and AIDS (Prevention and Control) Aact, 2008:

Section 15

1. *Every person residing in Tanzania may on his own motion volunteer to undergo HIV testing.*
2. *A child or a person with inability to comprehend the result may undergo HIV testing after a written consent of a parent or recognised guardian.*

Law of the Child Act 2009 [Cap 13], Section 4(1) A person below the age of eighteen years shall be known as a child.

Tanzanian law does not define 'mature minors'.

United Republic of Tanzania Ministry of Health and Social Welfare, National Aids Control Programme: Guidelines for HIV Counselling and Testing in Clinical Settings (July 2007) pg 18.

5.4.1 Minimum Age for HIV testing
PITC of children and adolescents below 16 years of age shall be carried out with the consent of parents or legal guardians, and when the health care practitioner has determined and is satisfied that testing is in the best interest of the child.

Adolescents aged 16 years and above may consent for PITC themselves. Also adolescents who are married, have children, or are sexually active shall be categorized as "mature minors" and permitted unrestricted access to PITC services irrespective of their age.

Age to report HIV status directly to adolescents

The law does not provide for a particular age which an adolescent's HIV status can be reported directly to him/her. However as seen in the legislation quoted, the test results are confidential and shall be shown only to the tested person with the exception of persons under 18 years of age, in which case the results are reported to the parent or guardian (this also applies to the mature minor - 16-17 years).

Legislation and policy framework on HIV testing

HIV and AIDS (Prevention and Control) Act, 2008:

Section 15, states that:

2. *A child or a person with inability to comprehend the result may undergo HIV testing after a written consent of a parent or recognised guardian.*

Section 16

1. *The results of an HIV test shall be confidential and shall be results released only to the person tested.*
2. *Notwithstanding subsection (1), the results of an HIV test maybe released to-*
   a. *in the case of a child, his parent or recognised guardian;*
   b. *in the case of person with inability to comprehend the results, his spouse or his recognised guardian*

Child Act 2009 (Chapter 13), states that a child is any person below the age of 18 years.

Chapter Five: Age of Consent on Antiretroviral Therapy

The National AIDS Policy of 2001 provides for everyone to have access to Antiretroviral Therapy (ART) since there are no explicit rules regarding the same, nor is there any specification as to the age of the person or the consent required. ART is still a broad concept in the United Republic of Tanzania, the only recognition the law gives it is Antiretroviral Drugs which can be taken from the moment the patient tests HIV positive. In the absence of legal prohibitions, young persons of any age may access ART, although there are no rules to prevent healthcare providers from requiring parental consent.

There is no Age of Consent to general medical treatment. Therefore, the study could not ascertain what applies to ART.

Legislation and policy framework on ART

The HIV and AIDS (Prevention and Control) Act, 2008, Section 24(2) reads:
The Ministry shall, where resources allow, take necessary steps to ensure the availability of ARVs and other healthcare services and medicines to persons living with HIV and AIDS and those exposed to risk of HIV infection.

Section 52
The Minister may make regulations prescribing
b. methods, programmes and coordination of distribution and use of ARVs.

Section 33
1. Any person living with HIV and AIDS shall using available resources have -
  a. a right to the highest attainable standard of physical and mental health; and
  b. a right to treatment of opportunistic infections.

Section 34
1. Every local government authority shall design, formulate, establish and coordinate mechanisms and strategic plans for ensuring that the most vulnerable children within its respective area are afforded means to access education, basic health care and livelihood services.

National AIDS Policy of 2001 *(page 5) states that:*
People Living with HIV and AIDS have the right to comprehensive healthcare and other social services, including legal protection against all forms of discrimination and human rights abuse. However, PLHAS may be required to meet some of the cost of the Highly Active Antiretroviral Therapy (HAART).

National AIDS Control Programme

This is inconsistent with the Age of Consent to sexual intercourse. There is no general Age of Consent to medical treatment. There is no contradiction with the answers given and a follow-on intervention is unclear.

Chapter Six: Age of consent and access to Pre-exposure Prophylaxis (PrEP) and Post-exposure Prophylaxis (PEP)

HIV Pre-exposure Prophylaxis (PrEP)

Tanzania does not have laws or policies that prohibit access to PrEP.

Young people's access to PrEP

The law is silent on PrEP, however, all HIV/AIDS infected young people should be treated as per the rights and obligations that they are entitled to, which also includes means to access education, basic healthcare and livelihood services among others.

Since there is no explicit rule with regard to the Age of Consent to access PrEP, we can conclude that the default legal position would permit all people living with HIV, to access PrEP regardless of their age although, in practice, healthcare providers could require parental consent. The country does not have a set Age of Consent to general medical treatment. Therefore, we cannot ascertain that it would also apply to PrEP.

Legislative and policy framework

The existing legislative and policy framework to access PrEP.

HIV and AIDS (Prevention and Control) Act, 2008
Section 33.

Post-exposure Prophylaxis (PEP)

The law does not specifically provide for the prohibition of PEP and it is mentioned in relation to the way an infected person is expected to be treated.

Legislation and policy framework

The HIV and AIDS (Prevention and Control) Act, 2008 articulates the legal and policy framework for one to access PrEP and PEP:

HIV and AIDS (Prevention and Control) Act, 2008, Section 12 states that; Every health practitioner shall during surgical, dental and similar procedures

Have a duty to ensure that-
2. A person who is the owner, manager or the in charge of healthcare facility shall have a duty to ensure-
a. the provision of Post-exposure Prophylaxis.

Section 26
A health practitioner who is exposed to or infected with HIV in the course of rendering healthcare services to a person living with HIV/AIDS shall be entitled to-
a. Post-exposure Prophylaxis.

Legislation and policy framework on access to PEP

The law is silent as to the rights of young people's access of PEP. Since there is no explicit rule, we can conclude that the default legal position would permit all people living with HIV, to access PEP regardless of their age although healthcare providers could, in practice, require parental consent.

The HIV and AID (Prevention and Control) Act, 2008:

Sections 12 and 26: There is no Age of Consent to general medical treatment. Therefore, we cannot ascertain that it would also apply to PEP. The law mentions PEP, but we do not have a specific policy or legislation dedicated solely to the provision of PEP and its issues therein.

Chapter Seven: Age of Consent and access to safe abortions and or postabortion care

Abortion is illegal in Tanzania and safe abortions are only allowed in instances of saving the pregnant mother's life.

Legislation and policy framework on safe abortion and postabortion care

In Tanzania, the Penal Code Chapter 16 RE: 2002 articulates the country's positon that safe abortions are permitted to save the pregnant mother's life.

The Penal Code Chapter 16 RE: 2002 Section 150 states, any person with intent to procure miscarriage of a woman by administering anything unlawfully leading to miscarriage.

Section 152 *also states that any person who supplies or procures to supply to a woman anything unlawful for miscarriage.*

Section 230:
A person is not criminally responsible for performing, in good faith and with reasonable care and skill, a surgical operation upon any person for his benefit, or upon an unborn child for the preservation of the mother's life if the performance of the operation is reasonable, having regard to the patient's state at the time and to all the circumstances of the case.

At the time of the review, the Government of Tanzania was expected to pass a new regulation to address access to safe abortions and postabortion care.

Chapter Eight: Age of Consent on access to Antenatal Care (ANC)

The law in Tanzania is silent on the Age of Consent on Antenatal Care (ANC). There are no explicit rules to address the issues. The study can conclude that every pregnant woman is not legally prevented from ANC regardless of her age although, in practice, healthcare providers could require parental consent for young women to access ANC. Furthermore with no specific Age of Consent to general medical treatment, the study could not verify or ascertain what applies to ANC.

Legislation and policy framework on ANC

ANC is accounted for in Tanzania's policy document implementing the Government's aim to reduce maternal deaths. Furthermore, the law stipulates that pregnant women with HIV should receive care and treatment while giving birth to reduce HIV transmission from mother to child. They, along with breastfeeding women, should receive counselling services to help prevent mother-to-child transmission of HIV. The Ministry of Health and Social Welfare: The National Roadmap Strategic Plan to accelerate the reduction of Maternal, Newborn, and Child Deaths in Tanzania.

Chapter Nine: Access to HPV vaccines and cervical cancer screening and treatment

At the time of the review, the United Republic of Tanzania had yet to draft a policy on this subject and due to the absence of any explicit rules for the same, the legal framework relies on the development of the National Cervical Cancer Control Strategy that plans on introducing Human Papillomavirus (HPV) vaccines to school girls, therefore omitting the motion of disabling access to HVP vaccines to people of any age.

As there is no Age of Consent to general medical treatment, the reviewers could not ascertain that it would also apply to HPV vaccines or cervical screening. A vaccination was about to be tested at the time of review among school girls in Tanzania but barriers exist as explained below.

Legislation and policy framework

Tanzania has one of the highest incidences of cervical cancer globally and at the time of the review was developing a National Cervical Cancer Control Strategy to introduce HPV vaccines to school girls. However, policymakers in Tanzania require information on the most-suitable target population for vaccination and the feasibility of different school-based delivery strategies and data on the acceptability and cost effectiveness of HPV vaccination.

The Mwanza Intervention Trials Unit (www.mitu.or.tz), a collaborative platform with the Tanzania National Institute for Medical Research (NIMR), and London School of Hygiene and Tropical Medicine (LSHTM) works on improving health through the development and evaluation of interventions against HIV and other health problems by conducting research, including clinical trials, to the highest international standards; to enhance the capacity to carry out such research in Tanzania.

Chapter Ten: Recommended intervention on legal and policy framework

Area	Category of regulation	Required intervention
Age of sexual intercourse	L	LR
Definition of statutory rape	L	LR
Exceptions on Age of Consent - For example 'gay sex'	L	LR
Young people's access to contraceptive services	S	N/A
Young people's access to emergency contraceptives	N/A	N/A
Policy framework and legislation on access to Antiretroviral Therapy (ART)	L	N/A
Policy and legislation on young people's access to PEP	L	N/A
Policy and legislation on young people's access to PreP	L	Minister to Sign Off New Practice
Policy framework and access to Antenatal Care (ANC)	L	N/A
Policy framework and legislation on access to HPV vaccines and cervical cancer screening and treatment	N/A	N/A
Policy framework and/or legislation on access to safe abortions and/or postabortion care	L	N/A

Legislation L | Policy P | Government To Pass New Regulations GR | Law Reform LR | Survey S

Chapter Ten: Recommended intervention on legal and policy framework (cont.)

Area	Category of regulation	Required intervention
Age of consent to access HIV testing without parental consent	N/A	N/A
Legal and policy framework on the Age of Consent where HIV status will be reported directly to an adolescent	L	GR
Addressing various policy and legislation inconsistencies	P	GR
Age at which HIV status will be reported directly to an adolescent and legal/policy requirements to report status to her/his parents?	L	N/A

Legislation L Policy P Government To Pass New Regulations GR Law Reform LR Survey S

Annex

KEY QUESTIONS FOR THE LEGAL REVIEW

1. At what age may sex between consenting individuals legally take place? (Age of Consent to sexual intercourse) Indicate if there are different ages for males and females?
2. Is there a definition of statutory rape? Please define.
3. Are there exceptions to question (1)? For example gay sex?
4. At what age may a young person access contraceptive services including contraceptive commodities? Please specify with and without parental consent if the answers are different.
5. May a young person access emergency contraceptives (e.g. the ‘Morning-after pill’) At what age? Please specify if there are different ages with and without parental consent.
6. policy framework and legislation enabling or disenabling access to Antiretroviral Therapy (ART), including Age of Consent, with and without parental consent.
7. Specify whether there is any prohibition on HIV Post-exposure Prophylaxis (PEP), including Age of Consent with and without parental consent.
8. If there is no prohibition in question 7, would young people be legally able to access PEP if it was offered? At what ages? Please specify if there are different ages with and without parental consent.
9. Is there any legislation or policy specifically enabling PEP use in country? Please specify if it deals with Ages of Consent and give details.
10. Specify whether there is any prohibition on HIV Pre-exposure Prophylaxis (PrEP), including Age of Consent with and without parental consent.
11. If there is no prohibition in question 10, would young people be legally able to access PrEP if it was offered? At what ages? Please specify if there are different ages with and without parental consent.
12. Is there any legislation or policy specifically enabling PrEP use in the country? Please specify if it deals with Ages of Consent and give details .
13. Please state whether there is policy framework and/or legislation enabling or disenabling access to safe abortions and/or postabortion care, including Age of Consent, with and without parental consent.
14. policy framework and legislation enabling or disenabling access to Antenatal Care (ANC), including Age of Consent with and without parental consent.
15. policy framework and legislation enabling or disenabling access to HPV vaccines and cervical cancer screening and treatment, including Age of Consent, with and without parental consent.
16. What is the Age of Consent to access HIV testing without parental consent?
17. What is the Age of Consent at which the HIV status will be reported directly to an adolescent and legal/policy requirements to report the status to her/his parents?
18. Please explain any inconsistencies between the answers above.

SRHR Africa Trust

GENDER EQUAL YOUTH HEALTH

www.satregional.org

@SATregional

SATregional
