# Supplementary material for: A Decision Aid for Postpartum Adolescent Family Planning: A Quasi-Experimental Study in Tanzania
Source: Int J Environ Res Public Health. 2023 Mar 10;20(6):4904. doi: 10.3390/ijerph20064904 (PMC10049540; doi:10.3390/ijerph20064904)
Supplement: Supplementary file 1 [file ijerph-20-04904-s001.zip › File S7 Green Star-English.pdf]

What Can You Do to Prevent Rapid Repeat Pregnancy?

A "Green Star" Decision Aid for Postpartum Adolescent Mothers below 20 Years

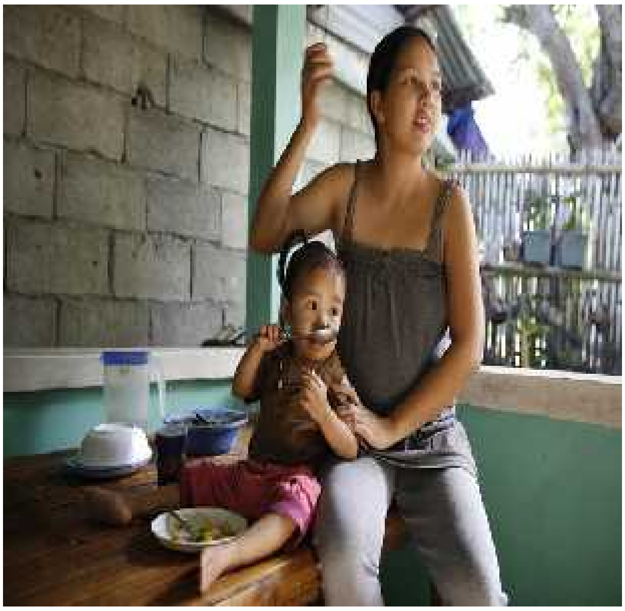

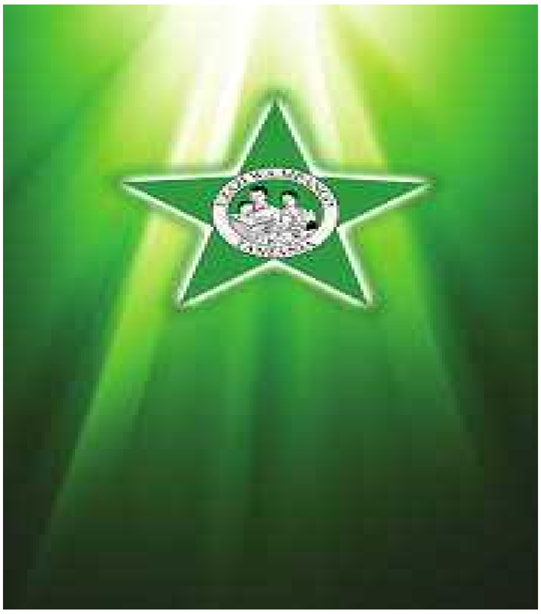

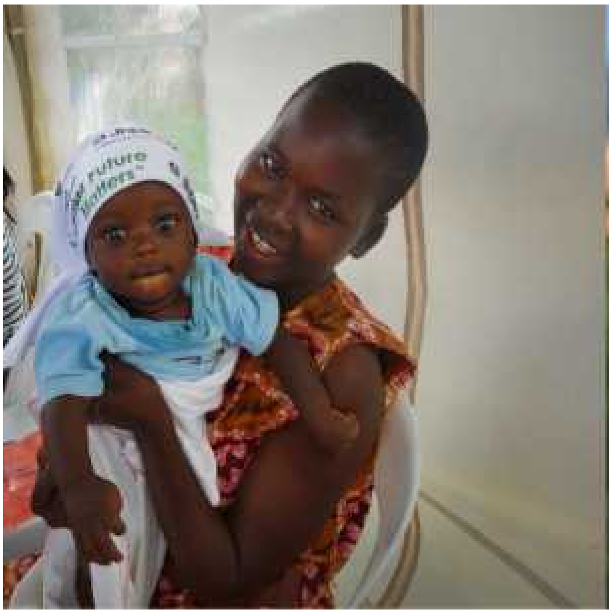

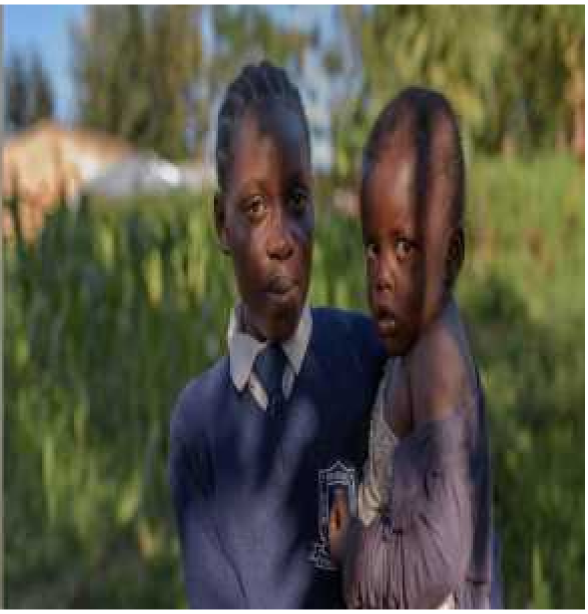

3rd Version

Step 1：Know how to make a decision with conviction

Preamble

About the family planning “Green Star” decision aid

i. This “Green Star” decision aid (DA) helps adolescents consider best option for the available long-acting reversible family planning methods immediately after birth

ii. We hope that information available in this DA will be of useful to help make the decision of your choice.

iii. The information is aiming at helping adolescent mothers to avoid getting unplanned rapid repeat pregnancy by providing information on the available long-acting reversible family planning (LARFP） option that you can get and use them before you are discharged from the hospital back home.

iv. The tool is designed specifically for adolescent mothers below age 20 years.

Note: The process of decision-making involves several steps. Nurse/midwife will guide you to make an informed decision about the option to use immediately after birth

Step 1：Know how to make a decision with conviction

Instructions on how to use this tool and "Your preferences"

We recommend that you …

1. Nurse/midwife together with the mother will reserve 40-50 minutes
2. HCP should have a pencil ready to use to mark your preferences as she explains in details about each option to you
3. Nurse/midwife together with the mother should read the entire decision aid, line after line and should not skip any section
4. At the end then choose your preferences on long-acting family planning methods.

Step 2: Understand the characteristics of the options

Introduction about Long-acting reversible family planning (LARFP) methods

- ✓ **Long-Acting Reversible Family Planning methods[1];**
    - are the most effective types of modern contraception
    - have a success rate of more than 99%
    - are very safe
    - prevents pregnancy for more than 3 years
    - are more effective than methods short-acting methods that rely on daily, weekly, or monthly use
    - can be removed any time if you want to get pregnant or stop using them
    - there are two types which are copper intrauterine devices and implants that includes Implanon and Jadelle

- ✓ **Who can use LARFP methods?[1]**

  Most women can use LARFP methods including women who;
    - Have or have not had children
    - Are married or are not married
    - Are of any age, including adolescents
    - Have just had an abortion or miscarriage (if no evidence of infection)
    - Are breastfeeding

Step 2: Understand the characteristics of the options

- ✓ **Intrauterine copper device (IUCD) and implants are described in details in the table below**

Table 1: Describing characteristics of IUCD and implants[1]

Option	IUCD	Implants
Image in situ	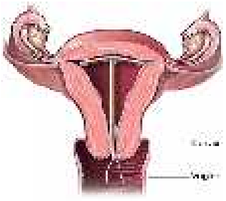	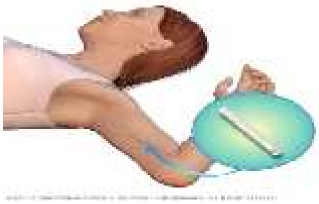
Place of insertion	Upper side of the **uterus**	Just under the skin, inner side of the none dominant upper arm
How it works	Weaken sperm motility	Prevents **ovulation**
Pregnancy prevention duration	10 years	Implanon 3 years<br>Jadelle 5 years
Type of method	None hormone	hormone (progesterone)

Step 2: Understand the characteristics of the options

Table 2: Benefits and side effects of IUCD and Implants

Benefits	IUCD	Implants
	Protective against endometrial cancer[2]	Reduces pain symptom for people with endometriosis[3]
	Safe for women whom hormones are contraindicated[1]	Safe for women whom estrogen hormone is contraindicated[1]
	It works as soon as it is put in place. No need for backup[1]	It works few days letter after insertion and it needs a 7 days backup[1]
	Fertility returns to normal as soon as the IUCD is taken out[4]	Fertility returns to normal as soon as the implant is taken out[5]
	Cannot be felt during sex, as threads will be trimmed to reduce its length[1]	It is virtually invisible, so a good option for those who want to keep it secrete[1]
**Side effects**	**IUCD**	**Implants**
	Heavy and prolonged bleeding in first few months[1,8]	Infrequent bleeding and amenorrhea[1,7]
	Little abdominal pain at time of insertion[1]	Little pain during insertion as pain relief medication will be used[1]
**Complications**	**IUCD**	**Implants**
	Perforation of the uterus but is rare (1 out 1,000 insertions)[10]	Infection at the insertion site but rare[1]

Step 2: Understand the characteristics of the options

Efficacy of each option

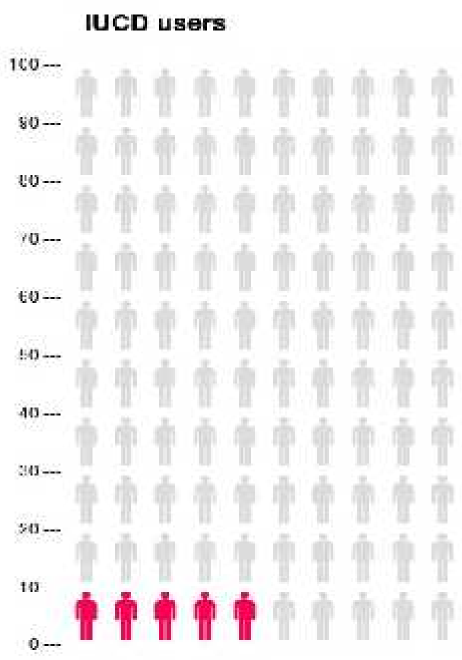

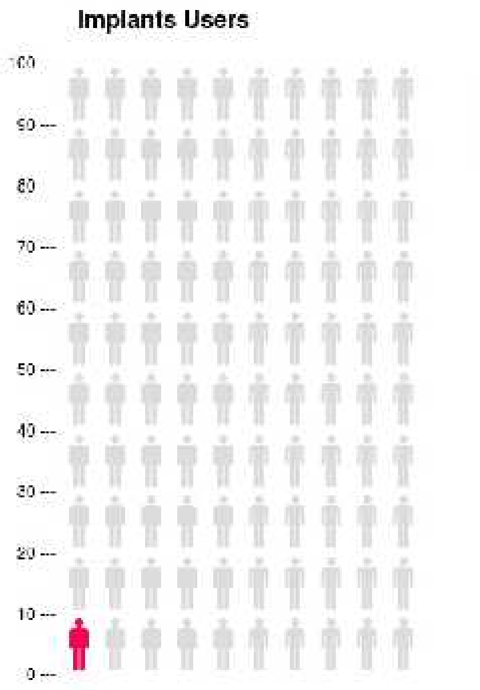

Out of **100** women who used IUCD, **5** had an unintended pregnancy[1]

Out of **100** women who used Implant, **1** had an unintended pregnancy[1]

Step 2: Understand the characteristics of the options

Satisfaction rate with each option

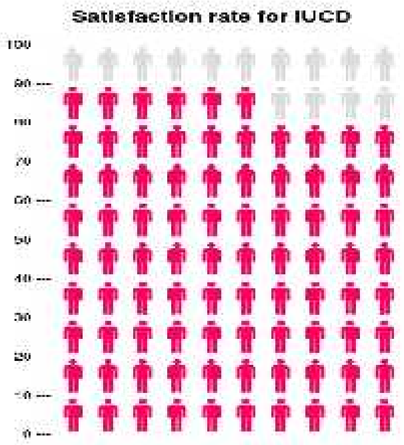

Out of 100 women who used IUCD, 86 were satisfied with the method[11]

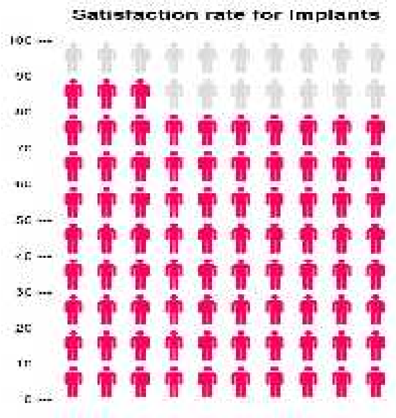

Out of 100 women who used Implants, 83 were satisfied with the method[11]

Fertility return within a year following removal of each option

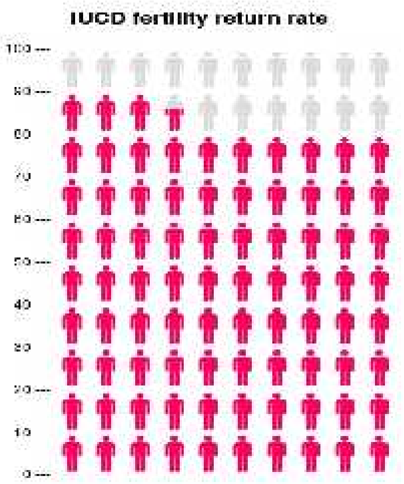

Out of 100 women, 83.6 conceived within a year following IUCD removal[4]

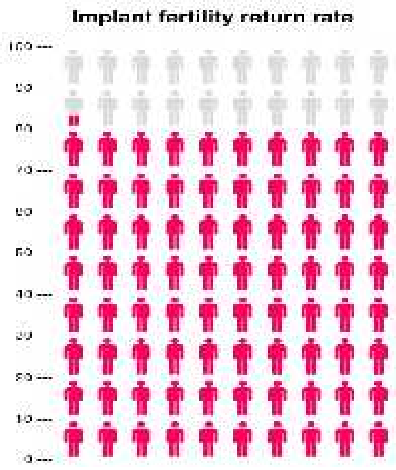

Out of 100 women, 80.3 conceived within a year following Implant[5] removal

Step 2: Understand the characteristics of the options

Effect of LARFP methods vs. short acting reversible family planning methods in preventing unwanted subsequent pregnancies

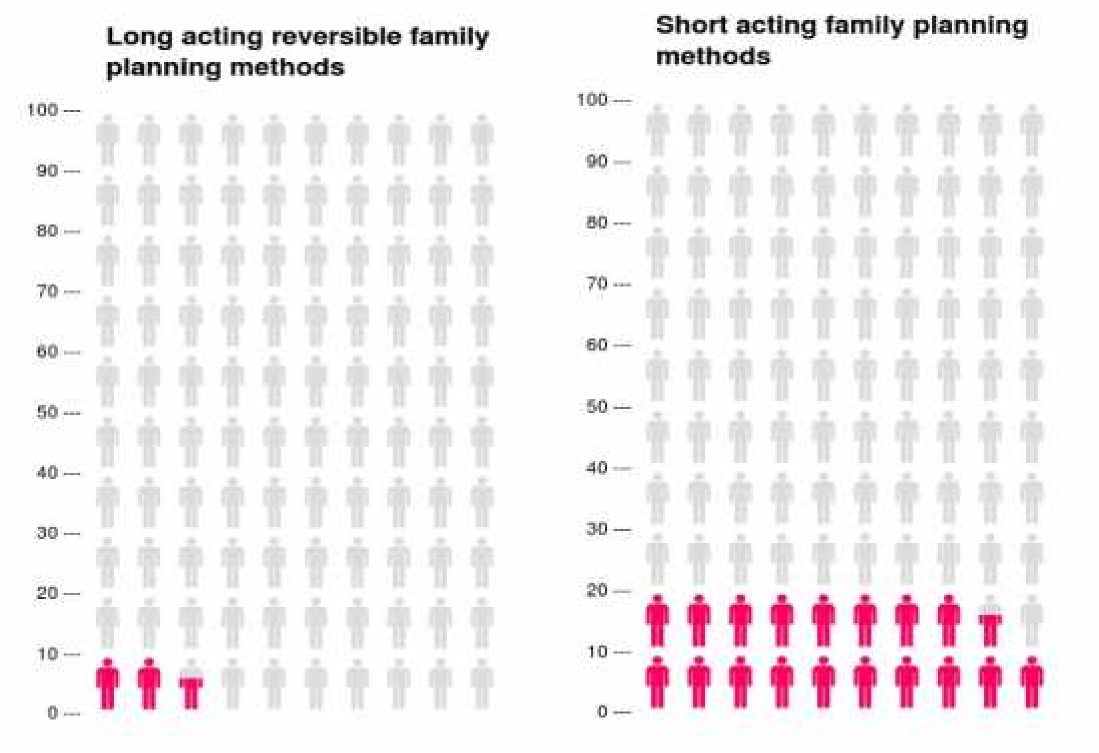

Out of **100** women who used long acting reversible family planning methods, **2.6** had an intended pregnancy within a year[12]

Out of **100** women who used short acting family planning methods, **18.6** had an intended pregnancy within a year[12]

Step 3: Clarify what is important to you

Level of importance of using LARFP immediately after birth

	Very important (0)	I do not know (2)	Not Important (4)
How important is it for you to prevent next pregnancy?			
Concern about the side effects of LARFP			
Type of partner's sexual preference			
Future plan for the next pregnancy			

Step 4: Make the decision[13]

	Yes (1)	No (0)
Do you feel SURE about the best choice for you?		
Do you know the benefits and risks of each option?		
Are you clear about which benefits and risks matter most to you?		
Do you have enough support and advice to make a choice?		

	**Check √ one**
I have decided to take **one option** soon after I deliver.	
I need to discuss the options with my important person(s). List____	
I need to read more about my option	
Other, please specify________________________________	

Now think about which option you are going to choose

Which option do you prefer? **Check √ one:**

Take the IUCD

Take the Implants

I do not know

Note:

If you have, any question write down here and ask a health care provider for clarification

Development process of the decision aid

This decision aid was created based on Ottawa Personal Decision Guide (2015) and opinions of doctors, nurses, midwives. Aid does not cover all medical information, but it contains information that you should know. There is no conflict of interest

Update information

This information is intended to facilitate communication with healthcare professionals in your decision-making and to help organize thoughts about your decision. It is not a substitute for medical advice.

References

1. World Health Organization Department of Reproductive Health and Research (WHO/RHR) and Johns Hopkins Bloomberg School of Public Health/Center for Communication Programs (CCP), Knowledge for Health Project. Family Planning: A Global Handbook for Providers (2018 update). Baltimore and Geneva: CCP and WHO, 2018.

2. Hubacher D & Grimes DA. Non-contraceptive health benefits of intrauterine devices: a systematic review. Obstet Gynecol Surv. 2002 Feb; 57(2):120-8. https://www.ncbi.nlm.nih.gov/pubmed/11832788. [Accessed 13th August, 2019]

3. Yisa SB, Okenwa AA & Husemeyer RP. Treatment of pelvic endometriosis with etonogestrel subdermal implant (Implanon®). J Fam Plann Reprod Health Care 2005; 31(1): 67–70. https://doi.org/10.1783/0000000052972799

4. Soeprono R. Return to fertility after discontinuation of copper IUD use: a study of 55 pregnancies involving Multiload Cu-250 users among private patients in Indonesia. Adv Contracept. 1988 4(2):95-107. https://www.ncbi.nlm.nih.gov/pubmed/3213675. [Accessed 25th July, 2019]
5. Buckshee k., Chatterjee P., et al. Return of fertility following discontinuation of Norplant-II subdermal implants: ICMR task force on hormonal contraception. Vol. 51 (4): 237-242. https://doi.org/10.1016/0010-7824(95)00039-D
6. Funk S, Miller MM, Mishell DR Jr, Archer DF, Poindexter A, Schmidt J, Zampaglione E. Safety and efficacy of Implanon, a single-rod implantable contraceptive containing etonogestrel. Contraception. 2005; 71(5):319-26. https://doi.org.10.1016/j.contraception.2004.11.007

7. Zheng SR, Zheng HM, Qian SZ, Sang GW, Kaper RF. A randomized multicenter study comparing the efficacy and bleeding pattern of a single – rod (Implanon) and a six-capsule (Norplant) hormonal contraceptive implant. Contraception 1999; 60: 1-8. https://doi.org/10.1016/S0010-7824(99)00053-0

8. Gabriel IL, Tudorache Set al. Birth Control and Family Planning Using Intrauterine Devices (IUDs)(2017). http://dx.doi.org/10.5772/intechopen.72242

9. Committee on Adolescent Health Care Long-Acting Reversible Contraception Working Group, the American College of Obstetricians and Gynecologists. Committee opinion no. 539: adolescents and long-acting reversible contraception: implants and intrauterine devices. Obstetrics and gynecology. 2012; 120(4): 983-988. https://m.acog.org/Clinical-Guidance-and-Publications/Committee-Opinions/Committee-on-Adolescent-Health-Care/Adolescents-and-Long-Acting-Reversible-Contraception. [Accessed 5 July, 2019]

10. WHO Scientific Group on the Mechanism of Action Safety and Efficacy of Intrauterine Devices & World Health Organization. (1987). Mechanism of action, safety and efficacy of intrauterine devices: report of a WHO Scientific Group [meeting held in Geneva from 1 to 4 December 1986]. World Health Organization. https://apps.who.int/iris/handle/10665/38182. [Accessed 4 August, 2019]

11. Peipert JF, Zhao Q, Allsworth JE, Petrosky E, Madden T, Eisenberg D, Secura G. Continuation and Satisfaction of Reversible Contraception. Obstet Gynecol. 2011: 117(5): 1105–1113. https://doi.org/10.1097/AOG.0b013e31821188ad.

12. Balwin MK, Edelman AB. The Effect of Long-Acting Reversible Contraception on Rapid RepeatPregnancy in Adolescents: A Review (2012). Journal of Adolescent Health 52 (2013) S47eS53. https://www.jahonline.org/article/S1054-139X(12)00715-X/pdf. [Accessed May, 2019]

13. Légaré F, Kearing S, Clay K, Gagnon S, D'Amours D, Rousseau M, & O'Connor A (2010). Are you SURE? Assessing patient decisional conflict with a 4-item screening test. Canadian family physician Medecin de famille canadien, 56(8), e308–e314.

Development of the tool was initiated and developed by Mushy Stella Emmanuel in collaboration with Horiuch Shigeko and Eri Shishindo

The project is funded by the Japan Society for the Promotion of Science (JSPS) Core to core program, Asia- Africa Science Platforms (2018-2021)

©St. Luke’s International University
