# Supplementary material for: A Decision Aid for Postpartum Adolescent Family Planning: A Quasi-Experimental Study in Tanzania
Source: Int J Environ Res Public Health. 2023 Mar 10;20(6):4904. doi: 10.3390/ijerph20064904 (PMC10049540; doi:10.3390/ijerph20064904)
Supplement: Supplementary file 1 [file ijerph-20-04904-s001.zip › File S8 Green Star-Kiswahili.pdf]

UTAFANYA NINI KUZUIA MIMBA ZA KARIBU KARIBU?

Muongozo wa Maamuzi ya Matumizi ya Nyota ya Kijani kwa Vijana Wanawake Wenye Umri Chini ya Miaka 20 Mara tu Baada ya Kujifungua

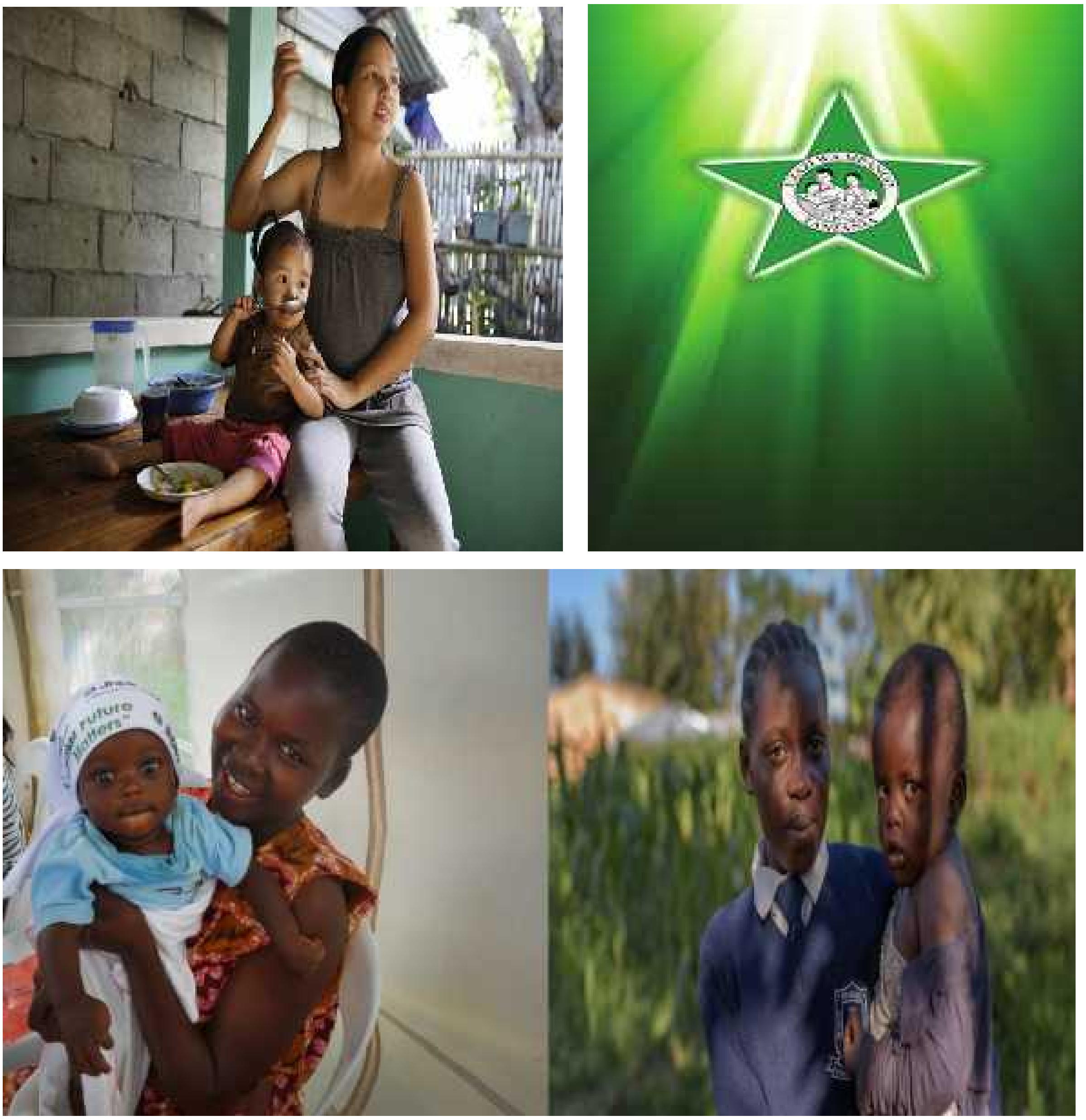

Hatua 1: Fahamu jinsi ya kufanya uamuzi kwa kusadikika

Utangulizi

Kuhusu Muongozo wa Maamuzi ya Nyota ya Kijani

i. Muongozo huu unaelekeza jinsi ya kufanya maamuzi sahihi katika kuchagua njia ya uzazi wa mpango ya muda mrefu isiyo ya kudumu mara baada ya kujifungua

ii. Taarifa zinazopatikana kwenye zana hii zitamsaidia mama mwenye umri chini ya miaka ishirini kutokupata mimba za karibu karibu kwa kumpa taarifa ya njia za uzazi wa mpango za muda mrefu zisizo za kudumu kabla hujaruhusiwa kwenda nyumbani baada ya kujifungua.

iii. Nimatumaini yetu kwamba taarifa zilizopo huku zitamsaidia mama kuchagua njia aipendayo ya uzazi wa mpango. Muongozo huu umetengenezwa kuwalenga akinamama mabinti wenye umri chini ya miaka 20.

Note: Mchakato mzima wa kuelekea kufanya maamuzi kuhusu njia gani ya uzazi wa mpango ya kutumia unahusisha hatua kadhaa. Mhudumu wa afya atakusomea kwa umakini hatua kwa hatua, neno kwa neno, mstari kwa mstari ili uwe kwenye nafasi nzuri ya kufanya maamuzi.

Hatua ya 1: Fahamu jinsi ya kufanya uamuzi kwa kusadikika

Maelezo ya jinsi ya kutumia zana hii na “mapendeleo yako”

Tunapendekeza kwamba …

1. Mhudumu wa afya na mama watalazimika kutenga dakika 40 hadi 50 za muda wao
2. Ni vema kuwepo na penseli tayari kuweka alama kwenye chaguo la njia itakayotumika
3. Someni njia zote kwa pamoja kuweza kufanya maamuzi mstari hadi mstari na msiruke sehemu yeyote.
4. Chagua “njia ya uzazi wa mpango ya muda mrefu isiyo ya kudumu utakayopenda kuitumia mara tu baada ya kujifungua – chaguo lako” mwishoni kabisa

Hatua 2: Fahamu sifa za chaguzi

Utangulizi kuhusu njia za uzazi wa mpango zisizo za kudumu za muda mrefu

✓ **Njia za Uzazi wa Mpango za Muda Mrefu zisizo za Kudumu[1];**

- Ni aina bora za njia za uzazi wa mpago za kisasa
- Zinafanya kazi kwa mafanikio ya zaidi ya asilimia tisini na tisa (99%)
- Ni salama
- Zinazuia mimba kwa zaidi ya miaka 3
- Zinafanya kazi vizuri kuliko njia za muda mfupi kama zile zinazotumika kwa kila siku, wiki au kila mwezi.
- Unaweza kutoa wakati wowote unapotaka kupata ujauzito au unapotaka kuacha kutumia
- Kuna aina mbili za njia za muda mrefu zisizo za kudumu ambazo ni Kitanzi/lupu na vipandikizi (Implanon na Jadelle)

✓ **Nani anaweza kutumia njia hizi za uzazi wa mpango za muda mrefu zisizo za kudumu[1]?**

Wanawake wengi wanaweza kutumia njia hizi wakiwemo wale ambao;

- Wanao au hawana watoto
- Wameolewa au hawajaolewa
- Wenye umri wowote pamoja na vijana
- Ujauzito umetoka au umetolewa
- Wananyonyesha

Hatua 2: Fahamu sifa za chaguzi

Sifa zinazoelezea kila Chaguo

- ✓ **Lupu/Kitanzi (IUCD) na Kipandikizi Zimeelezewa kwa Undani Zaidi Hapa Chini**

Jedwali la kwanza: Linaelezea Sifa za Lupu/Kitanzi na Kipandikizi[1]

Aina ya njia	Lupu/Kitanzi	Kipandikizi
Inavyoonekana ikiwa ndani	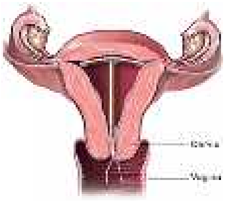	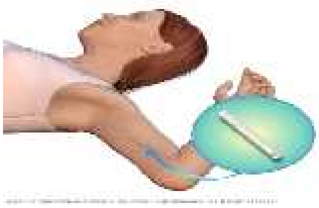
Inapowekwa	Kwa upande wa juu wa mji wa mimba	Chini ya ngozi katika mkono usioutunia sana
Inavyofanya kazi	Inadhoofisha mbegu za kiume ambazo ziko ndani ya uke kufikia yai lililokwisha pevuka	Inazuia yai kupevushwa/kupevuka
Muda wa kuzuia mimba	Miaka 10	Kipandikizi cha kijiti kimoja miaka 3<br>Kipandikizi cha vijiti viwili miaka 5
Aina ya njia	Haina kichocheo/homoni	Ina kichocheo/homoni (projesteroni)

Hatua 2: Fahamu sifa za chaguzi

Jedwali la Pili: Faida na Madhara ya Lupu na Kipandikizi

Faida	Lupu/Kitanzi	Kipandikizi
	Inazuia saratani ya kizazi[2]	Inapunguza maumivu hasa kwa watu wenye endometriosis[3]
	Ni salama kwa wanawake ambao homoni zinawaletea matatizo[1]	Ni salama kwa wanawake ambao kichocheo/homoni ya istrojeni inawaletea matatizo[1]
	Inafanya kazi punde tu inapowekwa kwenye kizazi na aihitaji njia mbadala[1]	Inanya kazi siku saba baada ya kuwekwa na utaitaji njia mbadala siku hizo za mwanzo[1]
	Hali ya kushika ujauzito inarudi punde baada ya kuitoa[4]	Hali ya kushika ujauzito inarudi punde tu baada ya kuitoa[5]
	Haiwezi kuhisiwa wakati wa kufanya tendo la ndoa maana nyuzi zake zinapunguzwa urefu wake[1]	Haiwezi kuonekana kwa macho hivyo ni nzuri kwa wale wanaotaka kuficha siri ya ni njia gani anatumia[1]
**Maudhi madogo madogo**	**Lupu/Kitanzi**	**Kipandikizi**
	Kutokwa na damu nyingi zaidi ya kawaida kwa miezi michache ya mwanzo[1,8]	Kutokuwa na mzunguko mzuri wa hedhi au kukosa hedhi[1,7]
	Maumivu kidogo ya tumbo wakati wa kuiweka[1]	Maumivu kidogo wakati wa kuiweka hata hivyo dawa ya kutuliza maumivu itatumika[1]
	Haiongezi uzito[1]	Inaweza kuongeza uzito japo imeripotiwa kutokea kwa wachache[1]
**Hali Tata (Complications)**	**Lupu/Kitanzi**	**Kipandikizi**
	Kutoboa kizazi lakini ni mara chache sana (1 kati 1,000 zilizowekwa)[10]	Maambukizi katika sehemu iliyowekwa lakini mara chache sana[1]

Hatua 2: Fahamu sifa za chaguzi

Ufanisi wa Kila Njia

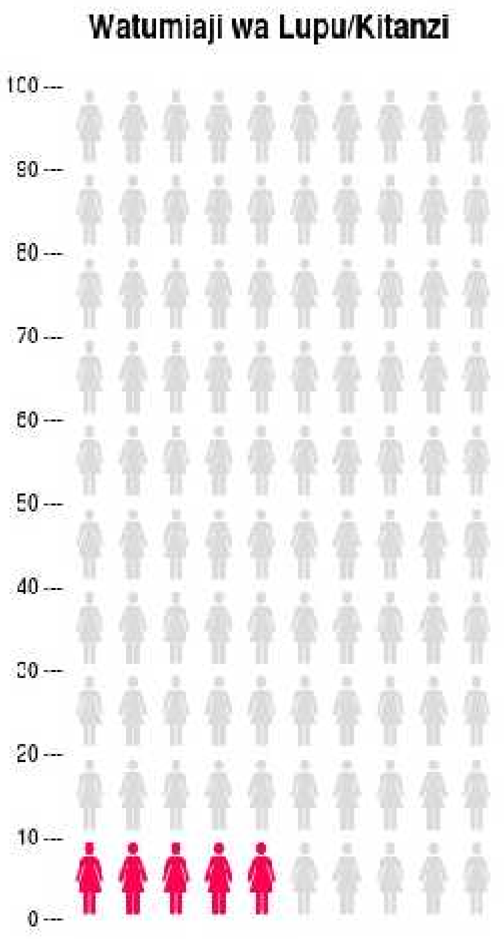

Kati ya wanawake **100** walioweka Lupu/Kitanzi; 5 pekee ndio walipata mimba zisizotarajiwa[1]

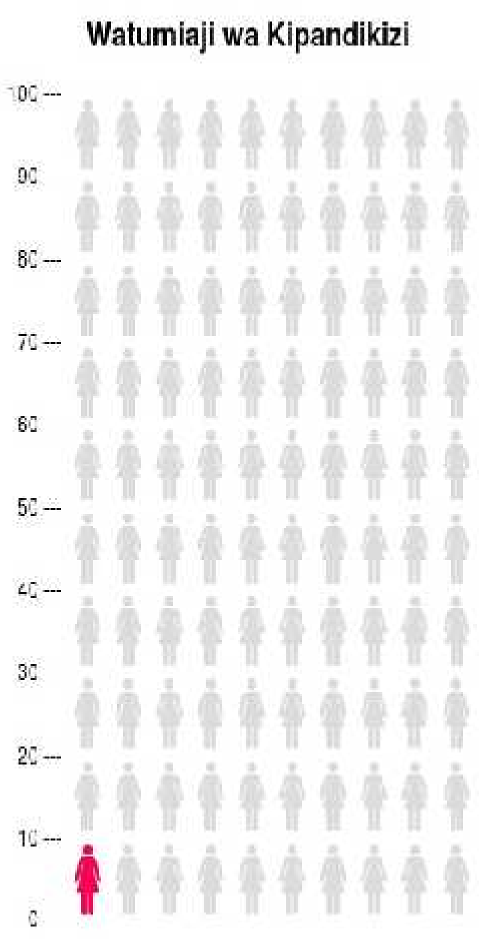

Kati ya wanawake **100** walioweka Kipandikizi; 1 pekee ndio alipata mimba zisizotarajiwa[1]

Hatua 2: Fahamu sifa za chaguzi

Kiwango cha Uradhi/Uridhishwaji kwa Kila Njia

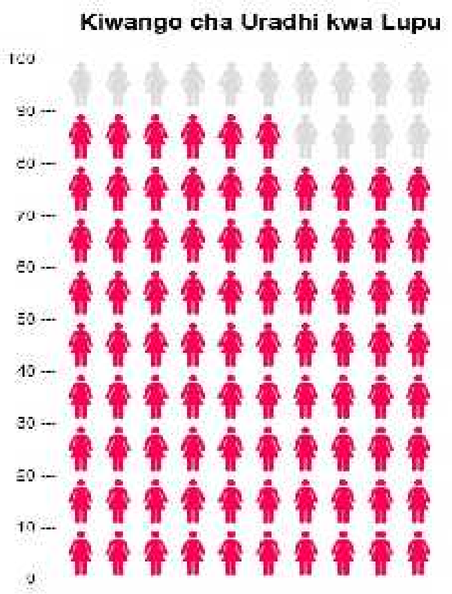

Kati ya Wanawake **100** waliotumia Lupu/Kitanzi, **86** waliridhishwa nayo[11]

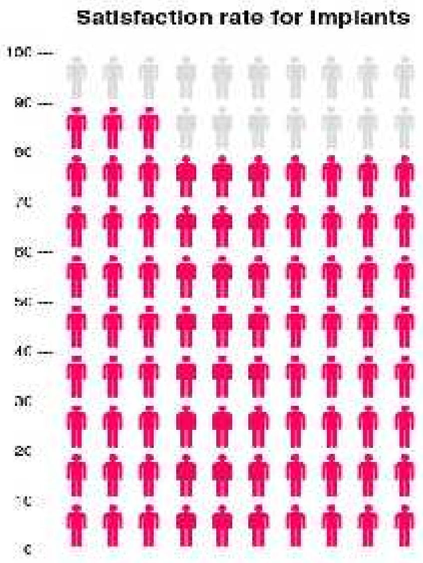

Kati ya Wanawake **100** waliotumia Kipandikizi, **83** waliridhishwa nayo[11]

Uwezo wa Kushika Mimba Ndani ya Mwaka Mmoja Baada ya Njia Kutolewa.

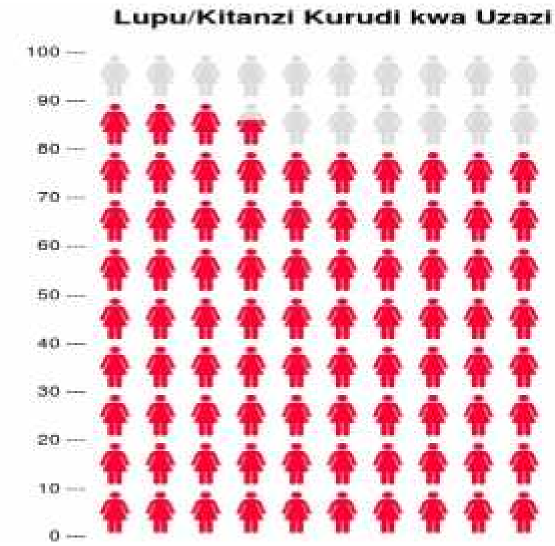

Kati ya Wanawake **100** waliotoa Lupu/Kitanzi, **83.6** walipata Ujauzito Ndani ya Mwaka Baada ya Kutoa[4]

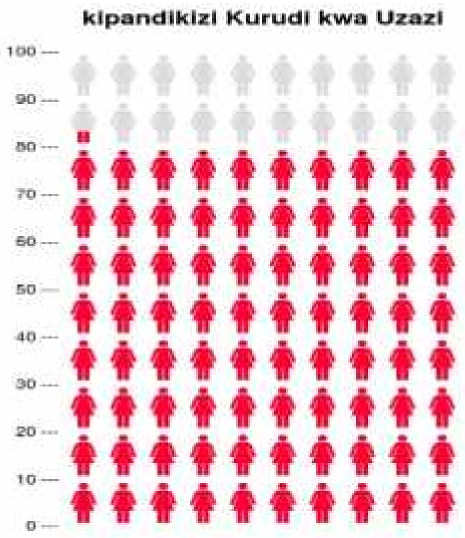

Kati ya Wanawake **100** waliotoa Lupu/Kitanzi, **80.3** walipata Ujauzito Ndani ya Mwaka Baada ya Kutoa[5]

Hatua 2: Fahamu sifa za chaguzi

Uwezo wa kuzuia mimba zisizotarajiwa kati ya njia za muda mrefu zisizo za kudumu na njia za muda mfupi

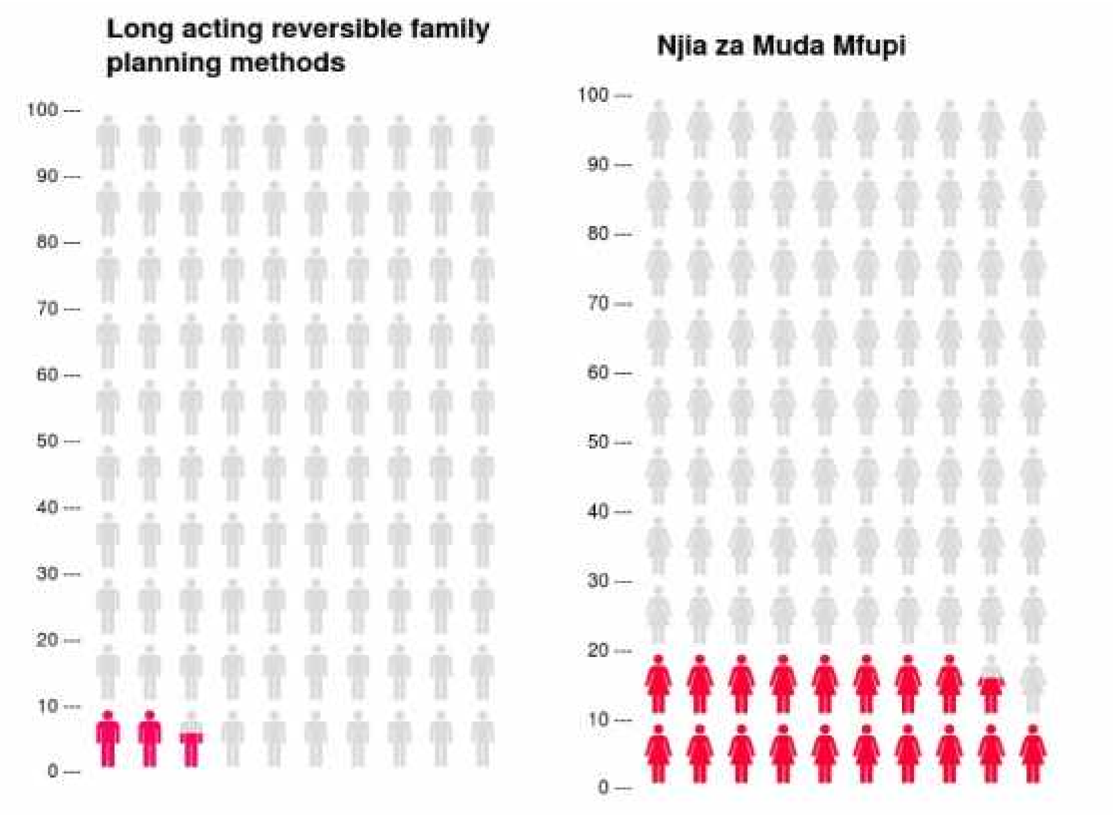

Kati ya wanawake **100** waliotumia njia za muda mrefu za uzazi wa mpango zisizo za kudumu, **2.6** walipata mimba isiotarajiwa ndani ya mwaka[12]	Kati ya wanawake **100** waliotumia njia za muda mfupi za uzazi wa mpango, **18.6** walipata mimba isiotarajiwa ndani ya mwaka[12]

Hatua 3: Fafanua yaliyo muhimu kwako

Kiwango cha Umuhimu wa Kutumia Njia za Muda Mrefu za Uzazi wa Mpango Zisizo za Kudumu Mara tu Baada ya Kujifungua

	Muhimu sana (0)	Sifahamu (2)	Sio muhimu (4)
Kuna umuhimu kiasi gani kuzuia mimba ifuatayo isitokee mapema?			
Wasiwasi juu ya athari za LARFP			
Upendeleo wa aina ya ngono wa mwenzi wako			
Mpango wa baadaye wa ujauzito ujao			

Hatua 4: Fanya uamuzi[13]

	**Ndiyo (1)**	**Hapana (0)**
Je! Unajisikia hakika juu ya Chaguo bora kwako?		
Je! Wajua faida na hatari ya njia uliyochagua?		
Je! Una uhakika juu ya faida na hatari gani muhimu kwako?		
Je! Una Msaada wa kutosha Kufanya Chaguo?		

	Weka √ kwenye mojawapo
Nimeamua kutumia njia mojawapo mara baada ya kujifungua	
Naitaji kuzungumza kuhusu uchaguzi sahihi na mtu wangu wa karibu (mtaje)________________________________	
Nahitaji kusoma zaidi	
Nyingine taja tafadhali ____________________________	

Sasa fikiria juu ya chaguo gani utachagua

Je unapendelea njia gani? Weka alama ya vyema √ kwenye mojawapo:

Lupu/Kitanzi

Kipandikizi

Sifahamu

Maoni:

Kama una maswali yaandike hapa na umuulize mtoa huduma kupatiwa ufafanuzi wake.

Mchakato uliotumika kutengeneza zana hii ya Nyota ya Kijani kukusaidia kufanya maamuzi

Hii zana ya Nyota ya Kijani ya kukusaidia kufanya maamuzi ilitengenezwa kwa kufuata muongozo uliowekwa na Ottawa Personal Decision Guide (2015) na maoni yaliyotoka kwa madaktari, wauguzi pamoja na wakunga. Muongozo huu auakisi taarifa zote za kiafya, lakini unazo taarifa za muhimu unazopaswa kujua. Hakuna mgongano wa kimaslahi kati ya watafiti wala wahudumu wa afya

Taarifa zilizoboreshwa

Taarifa hizi zinalenga kurahisisha mawasiliano kati ya watoa huduma wa afya katika kupangilia vyema maamuzi utakayoyachagua. Sio mbadala wa ushauri wa kiafya

References

1. World Health Organization Department of Reproductive Health and Research (WHO/RHR) and Johns Hopkins Bloomberg School of Public Health/Center for Communication Programs (CCP), Knowledge for Health Project. Family Planning: A Global Handbook for Providers (2018 update). Baltimore and Geneva: CCP and WHO, 2018.

2. Hubacher D & Grimes DA. Non-contraceptive health benefits of intrauterine devices: a systematic review. Obstet Gynecol Surv. 2002 Feb; 57(2):120-8. https://www.ncbi.nlm.nih.gov/pubmed/11832788. [Accessed 13th August, 2019]

3. Yisa SB, Okenwa AA & Husemeyer RP. Treatment of pelvic endometriosis with etonogestrel subdermal implant (Implanon®). J Fam Plann Reprod Health Care 2005; 31(1): 67–70. https://doi.org/10.1783/0000000052972799

4. Soeprono R. Return to fertility after discontinuation of copper IUD use: a study of 55 pregnancies involving Multiload Cu-250 users among private patients in Indonesia. Adv Contracept. 1988 4(2):95-107. https://www.ncbi.nlm.nih.gov/pubmed/3213675. [Accessed 25th July, 2019]

5. Buckshee k., Chatterjee P., et al. Return of fertility following discontinuation of Norplant-II subdermal implants: ICMR task force on hormonal contraception. Vol. 51 (4): 237-242. https://doi.org/10.1016/0010-7824(95)00039-D

6. Funk S, Miller MM, Mishell DR Jr, Archer DF, Poindexter A, Schmidt J, Zampaglione E. Safety and efficacy of Implanon, a single-rod implantable contraceptive containing etonogestrel. Contraception. 2005; 71(5):319-26. https://doi.org.10.1016/j.contraception.2004.11.007

7. Zheng SR, Zheng HM, Qian SZ, Sang GW, Kaper RF. A randomized multicenter study comparing the efficacy and bleeding pattern of a single – rod (Implanon) and a six-capsule (Norplant) hormonal contraceptive implant. Contraception 1999; 60: 1-8. https://doi.org/10.1016/S0010-7824(99)00053-0

8. Gabriel IL, Tudorache Set al. Birth Control and Family Planning Using Intrauterine Devices (IUDs)(2017). http://dx.doi.org/10.5772/intechopen.72242

9. Committee on Adolescent Health Care Long-Acting Reversible Contraception Working Group, the American College of Obstetricians and Gynecologists. Committee opinion no. 539: adolescents and long-acting reversible contraception: implants and intrauterine devices. Obstetrics and gynecology. 2012; 120(4): 983-988. https://m.acog.org/Clinical-Guidance-and-Publications/Committee-Opinions/Committee-on-Adolescent-Health-Care/Adolescents-and-Long-Acting-Reversible-Contraception. [Accessed 5 July, 2019]

10. WHO Scientific Group on the Mechanism of Action Safety and Efficacy of Intrauterine Devices & World Health Organization. (1987). Mechanism of action, safety and efficacy of intrauterine devices: report of a WHO Scientific Group [meeting held in Geneva from 1 to 4 December 1986]. World Health Organization. https://apps.who.int/iris/handle/10665/38182. [Accessed 4 August, 2019]

11. Peipert JF, Zhao Q, Allsworth JE, Petrosky E, Madden T, Eisenberg D, Secura G. Continuation and Satisfaction of Reversible Contraception. Obstet Gynecol. 2011: 117(5): 1105–1113. https://doi.org/10.1097/AOG.0b013e31821188ad.

12. Balwin MK, Edelman AB. The Effect of Long-Acting Reversible Contraception on Rapid RepeatPregnancy in Adolescents: A Review (2012). Journal of Adolescent Health 52 (2013) S47eS53. https://www.jahonline.org/article/S1054-139X(12)00715-X/pdf. [Accessed May, 2019]

13. Légaré F, Kearing S, Clay K, Gagnon S, D'Amours D, Rousseau M, & O'Connor A (2010). Are you SURE? Assessing patient decisional conflict with a 4-item screening test. Canadian family physician Medecin de famille canadien, 56(8), e308–e314.

Zana hii imetengenezwa na Mushy Stella Emmanuel kwa kushirikiana na Horiuch Shigeko na Eri Shishindo.

Mradi huu unafadhiliwa na Japan Society for the Promotion of Science (JSPS) Core to core program, Asia- Africa Science Platforms (2018-2021)

©St. Luke’s International University
